# Functional polymorphism of the renalase gene is associated with cardiac hypertrophy in female patients with aortic stenosis

**DOI:** 10.1371/journal.pone.0186729

**Published:** 2017-10-24

**Authors:** Ewa Orlowska-Baranowska, Lucja Gadomska vel Betka, Jaroslaw Gora, Rafal Baranowski, Ewa Pedzich-Placha, Dariusz Zakrzewski, Angelika Dlugosz, Helena Kossowska, Agnieszka Zebrowska, Ewelina Zakoscielna, Anna Janiszewska, Tomasz Hryniewiecki, Zbigniew Gaciong, Grzegorz Placha

**Affiliations:** 1 Department of Acquired Cardiac Defects, Institute of Cardiology, Warsaw, Poland; 2 Department of Internal Medicine, Hypertension, and Vascular Diseases, Medical University of Warsaw, Warsaw, Poland; 3 Department of Arrhythmia, Institute of Cardiology, Warsaw, Poland; Maastricht University, NETHERLANDS

## Abstract

Renalase decreases circulating catecholamines concentration and is important in maintaining primary cellular metabolism. Renalase acts through the plasma membrane calcium ATPase 4b in the heart, which affects pressure overload but not exercise induced heart hypertrophy. The aim of this study was to test the association between a functional polymorphism Glu37Asp (rs2296545) of the renalase gene and left ventricular hypertrophy in a large cohort of patients with aortic stenosis. The study group consisted of 657 patients with aortic stenosis referred for aortic valve replacement. Preoperative echocardiographic assessment was performed to obtain cardiac phenotypes. Generalized-linear models were implemented to analyze data using crude or full model adjusted for selected clinical factors. In females, the Asp37 variant of the Glu37Asp polymorphism was associated with higher left ventricular mass (p = 0.0021 and p = 0.055 crude and full model respectively), intraventricular septal thickness (p = 0.0003 and p = 0.0143) and posterior wall thickness (p = 0.0005 and p = 0.0219) all indexed to body surface area, as well as relative wall thickness (p = 0.001 and p = 0.0097). No significant associations were found among the male patients. In conclusion, we have found the association of the renalase Glu37Asp polymorphism with left ventricle hypertrophy in large group of females with aortic stenosis. The Glu37Asp polymorphism causes not only amino-acid substitution in FAD binding domain but may also change binding affinity of the hypoxia- and hypertrophy-related transcription factors and influence renalase gene expression. Our data suggest that renalase might play a role in hypertrophic response to pressure overload, but the exact mechanism requires further investigation.

## Introduction

Left ventricular hypertrophy (LVH) which develops in patients with aortic stenosis (AS) is associated with increased mortality and morbidity before and after aortic valve replacement. The hypertrophic response to pressure overload from AS is heterogeneous and may depend on many factors including gender and genetic predisposition[1]. The familial predisposition of LVH was found in many studies[2]. A number of genetic loci were identified in case-control and wide-genome association studies in the general population or in hypertensive patients. Most of the associations failed to replicate in other cohorts[3, 4] which may be due to the fact that the degree of LVH is low in those populations and each study requires a very large number of participants to gain statistical power. Marked LVH and its poor correlation with pressure load[5] makes patients with AS a perfect population to study the genetics of heart hypertrophy. In 2005 a new circulating protein expressed in the kidney and heart was discovered and named renalase[6]. Originally, it was suggested that renalase could possibly affect the cardiovascular system by catabolizing circulating catecholamines[7], yet the oxidative properties of the enzyme were recently questioned[8]. New data suggests that renalase is an intracellular molecule important in maintaining primary metabolism[9]. Recently the plasma membrane calcium ATPase 4b (PMCA4b) was identified as a receptor for extracellular renalase. The PMCA4b mediates renalase dependent cell signaling and cytoprotection, which are not related to intrinsic enzymatic activities of renalase[10]. Interestingly, PMCA4b affects pressure overload but not exercise induced heart hypertrophy[11]. The renalase gene (RNLS) is located on chromosome 10 at q23.31 and its polymorphisms were associated with essential hypertension, type 1 diabetes and stroke[12–15]. In subjects diagnosed with coronary artery disease (CAD) a functional missense polymorphism Glu37Asp (rs2296545) in RNLS gene was associated with increased risk of LVH, ventricular dysfunction, reduced exercise capacity and inducible ischemia[16]. We studied the association between the most common genetic variation in the RNLS locus resulting in glutamic to aspartic acid change at amino acid residue 37 (Glu37Asp; rs2296545; NM_001031709.2:c.111G>C) and LVH in a large cohort of patients with AS.

## Materials and methods

The study group consisted of 657 patients (387 men and 270 women) referred to the Department of Acquired Cardiac Defects at the Institute of Cardiology in Warsaw for surgical intervention due to severe AS over the years 1995 to 2006. Patients with coexisting mitral and/or tricuspid valve disease or moderate/severe aortic regurgitation were excluded from the study. This study was approved by the Human Ethics Committees of the Medical University of Warsaw (KB/106/2007, KB/74/2012, KB/119/A/2013) and the Institute of Cardiology (959, 1169, 1448) and conformed to the ethical guidelines of the 1975 Declaration of Helsinki. Written informed consent was obtained from all study participants. All patients with a history of angina or aged >50 years underwent coronary arteriography. Significant coronary artery disease was defined as a reduction of at least 70% in the diameter of a major coronary artery or a 50% reduction in the left main coronary artery diameter.

Echocardiographic examination and calculations were done as previously described[17]. Briefly, the left ventricular mass (LVM) was calculated according to the Penn Convention modified by Devereux LVM (g) = 1.04[(IVST + LVEDD + PWT)^3^—LVEDD^3^] - 13.6 where LVEDD is the left ventricular end-diastolic diameter, IVST is the intraventricular septal thickness in diastole, and PWT is the posterior wall thickness in diastole. The body surface area (BSA) was calculated using the modified DuBois formula: BSA = (height (m)^0.73^ × body weight (kg)^0.4^ × 71.84)/10,000. The relative wall thickness (RWT) was calculated as RWT = PWT/ (0.5) LVEDD. The left ventricular geometry was classified as proposed by Duncan et al.[18]: a) concentric hypertrophy, increased LVM/height and increased RWT; b) eccentric hypertrophy, increased LVM/height and normal RWT; c) concentric remodeling, normal LVM/height and increased RWT; and d) normal geometry, normal LVM/height and normal RWT. An LVM/height of 143 g/m or less for men and of 102 g/m or less for women and an RWT equal to at most 0.45 were considered normal.

DNA was isolated from frozen peripheral blood samples with QiaAmp DNA Mini Kit (Qiagen). The ViiA^™^ 7 Real-Time PCR System was used to perform genotyping of rs2296545 polymorphism using the 5’ nuclease allelic discrimination assay according to the manufacturer's protocol (TaqMan assay, Life Technologies, USA). The assay number C__15753060_10 was designed on forward strand, therefore reference allele C corresponded to G in renalase’s mRNA and finally Glutamic Acid (Glu) at amino acid residue 37 of the protein (since RNLS gene is located on reverse strand), consequently reference allele G corresponded to C in the mRNA sequence and Aspartic Acid (Asp) in the protein. Ten percent of randomly selected samples were done in duplicates.

We used a Supplementary Data Set 1. SNPs tested for imbalance in DNA accessibility, which can be retrieved from http://www.nature.com/ng/journal/v47/n12/extref/ng.3432-S5.txt [19]. Additional data from ENCODE is available from Gene Expression Omnibus (GEO) (GSE18927, GSE26328, GSE29692 and GSE55579 for DNase-seq data and GSE30263 for ChIP-seq data).

For the computational identification of transcription factor which binding sites are affected by rs2296545 a MAPPER2[20, 21] the platform that combines TRANSFAC[22] and JASPAR[23] motifs with the search power of profile hidden Markov models, was used (http://genome.ufl.edu/mapper/).

The Statistical analysis was performed as previously described[17]. The echocardiographic parameters were not Gaussian distributed and were therefore natural log-transformed before analysis[24]. We tested the departure from the Hardy-Weinberg equilibrium using Haploview 4.0[25]. The primary quantitative outcome variable of the association analyses was LVM/BSA and the principal explanatory variable was rs2296545. The SNP was coded into three classes (major allele homozygote = 0, heterozygote = 1 or minor allele homozygote = 2) and analyzed under an additive genetic model. If the test for additive model was significant at the 0.05 significance level, then the dominant and recessive models were examined to see if they significantly improve the fit. Generalized linear models (PROC GLM in SAS software version 9.4; SAS Institute Inc, Cary, NC, USA) were used to analyze the effects of genetic and clinical covariates on a continuous outcome. The crude model tested the effect of SNP only, but the full model included the following clinical variables selected in our previous work[17]: gender, age, ejection fraction and maximal aortic gradient. P-values < 0.05 were designated as significant. The mean values presented in the text are back-transformed to the original scale from the natural log-transformed data shown in tables. We used Quanto[26, 27] to estimate the power of a statistical test: the minor allele frequency of rs2296545 is 44.7%, the sample size is 657, the overall mean value of the natural logarithm of LVM/BSA is 5.8980 and standard deviation is 0.2903, at a significance level of 0.05 the power is ≥80% when the risk-associated SNP explains at least 4.5%, 6.9% or 8.0% of the variance in additive, dominant or recessive model respectively.

## Results

### Patient characteristics

The study group was described previously[17]. The demographic and echocardiographic data for patients with AS are summarized in Table 1.

**Table 1:** Demographic characteristics and echocardiographic data for patients with aortic stenosis.

	Female (N = 270)	Male (N = 387)	
	Mean ± SD	Mean ± SD	P
Age, y	63.83 ± 10.66	59.78 ± 10.39	<0.0001
Height, cm	160.16 ± 5.83	172.19 ± 5.86	<0.0001
Weight, kg	68.94 ± 12.60	77.28 ± 11.58	<0.0001
BMI, kg/m^2^	26.88 ± 4.82	26.06 ± 3.69	0.0312
EF, %	66.77 ± 11.89	60.56 ± 15.07	<0.0001
Maximal aortic gradient, mm Hg	103.91 ± 27.32	95.96 ± 27.47	0.0010
Mean aortic gradient, mm Hg	64.73 ± 18.66	58.95 ± 18.05	0.0007
LVM, g	325.42 ± 86.09	418.29 ± 117.02	<0.0001
LVM/BSA, g/m^2^	190.74 ± 52.89	221.01 ± 63.36	<0.0001
LVM/height, g/m	203.43 ± 54.54	243.09 ± 68.41	<0.0001
LVM/height^2.7^, g/m^2.7^	91.83 ± 26.42	96.93 ± 29.40	0.0198
LVEDD, mm	48.31 ± 6.03	53.91 ± 7.63	<0.0001
LVEDD/BSA, mm/m^2^	28.34 ± 4.19	28.51 ± 4.60	0.8917
LVEDD/height, mm/m	30.19 ± 3.86	31.32 ± 4.43	0.0040
IVST, mm	13.98 ± 2.25	14.71 ± 2.49	0.0001
IVST/BSA, mm/m^2^	8.21 ± 1.50	7.78 ± 1.44	<0.0001
IVST/height, mm/m	8.74 ± 1.46	8.56 ± 1.53	0.0435
PWT, mm	13.20 ± 1.88	13.83 ± 1.98	<0.0001
PWT/BSA, mm/m^2^	7.76 ± 1.36	7.31 ± 1.15	<0.0001
PWT/height, mm/m	8.25 ± 1.25	8.04 ± 1.19	0.0162
RWT	0.56 ± 0.11	0.52 ± 0.11	0.0006
(IVST+PWT), mm	27.17 ± 3.82	28.53 ± 4.15	<0.0001
(IVST+PWT)/BSA, mm/m^2^	15.97 ± 2.71	15.09 ± 2.44	<0.0001
(IVST+PWT)/height, mm/m	17.00 ± 2.52	16.60 ± 2.54	0.0222
Left ventricular geometry, N (%)			
concentric hypertrophy	232 (85.9%)	287 (74.1%)	0.0003
eccentric hypertrophy	38 (14.1%)	90 (23.3%)	0.0036
concentric remodeling	0 (0.0%)	9 (2.3%)	
normal geometry	0 (0.0%)	1 (0.3%)	
Hypertension, N (%)	127 (49%)	129 (35%)	
Diabetes, N (%)	13 (5%)	25 (6%)	
Significant coronary artery disease	44 (17%)	93 (25%)	
NYHA, N (%)			
I	11 (4%)	28 (7%)	
II	54 (21%)	107 (29%)	
III	113 (44%)	138 (37%)	
IV	71 (27%)	84 (22%)	

BMI, body mass index; BSA, body surface area; EF, ejection fraction; IVST, intraventricular septal thickness in diastole; LVEDD, left ventricular end-diastolic diameter; LVM, left ventricular mass; NYHA, New York Heart Association Heart Failure Classification; PWT, posterior wall thickness in diastole; RWT, relative wall thickness; P, p value for difference between men and women.

### Genotyping

Genotyping was successful for 99.38% of the samples. Concordance rate between replicates was as high as 100%. The genotypes distribution was consistent with Hardy-Weinberg equilibrium (p = 0.99).

### Associations between rs2296545 polymorphism and LVM/BSA

The analysis (Table 2) of all of the patients, identified a significant genetic association with high LVM/BSA for the Asp37 variant of rs2296545, and an additive genetic model was suggested (crude model, mean: 191.5 for Glu/Glu variant, 202.6 for Glu/Asp, and 209.3 for Asp/Asp, p = 0.0039). The association was moderately reduced by the adjustment for age, sex, ejection fraction, and maximal aortic gradient (full model, p = 0.0469).

**Table 2:** Association between functional SNP in the RNLS gene and natural logarithm of LVM/BSA.

	Mean (SE) [N]					
	Crude			Adjusted*		
	All	Female	Male	All	Female	Male
Tagging SNPs:						
rs2296545	Additive					
Glu/Glu	5.255 (0.020) [189]	5.155 (0.027) [88]	5.341 (0.027) [101]	5.265 (0.018) [187]	5.188 (0.025) [87]	5.344 (0.025) [100]
Glu/Asp	5.311 (0.014) [344]	5.226 (0.022) [134]	5.366 (0.018) [210]	5.296 (0.013) [340]	5.215 (0.020) [134]	5.370 (0.017) [206]
Asp/Asp	5.344 (0.025) [120]	5.296 (0.037) [48]	5.376 (0.032) [72]	5.324 (0.022) [120]	5.272 (0.033) [48]	5.376 (0.029) [72]
P_ADD_	0.0039	0.0021	0.3887	0.0469	0.0551	0.3905
rs2296545	Dominant					
Glu/Glu	5.255 (0.020) [189]	5.155 (0.027) [88]	5.341 (0.027) [101]	5.266 (0.018) [187]	5.188 (0.025) [87]	5.344 (0.025) [100]
Glu/Asp or Asp/Asp	5.320 (0.012) [464]	5.244 (0.019) [182]	5.368 (0.016) [282]	5.303 (0.011) [460]	5.230 (0.017) [182]	5.372 (0.015) [278]
P_DOM_	0.0064	0.0087	0.3908	0.0820	0.1761	0.3535
rs2296545	Recessive					
Glu/Glu or Glu/Asp	5.291 (0.011) [533]	5.198 (0.017) [222]	5.358 (0.015) [311]	5.285 (0.010) [527]	5.204 (0.015) [221]	5.362 (0.014) [306]
Asp/Asp	5.344 (0.025) [120]	5.296 (0.037) [48]	5.376 (0.032) [72]	5.324 (0.022) [120]	5.271 (0.033) [48]	5.376 (0.029) [72]
P_REC_	0.0606	0.0190	0.6135	0.1250	0.0725	0.6739

SE, standard error; N, number of individuals; BSA, body surface area; LVM, left ventricular mass; SNP, single nucleotide polymorphism; P_ADD_, p value for additive model; P_DOM_, p value for dominant model; P_REC_, p value for recessive model;

*Adjusted for gender, age, ejection fraction, maximal aortic gradient.

**Fig 1:**
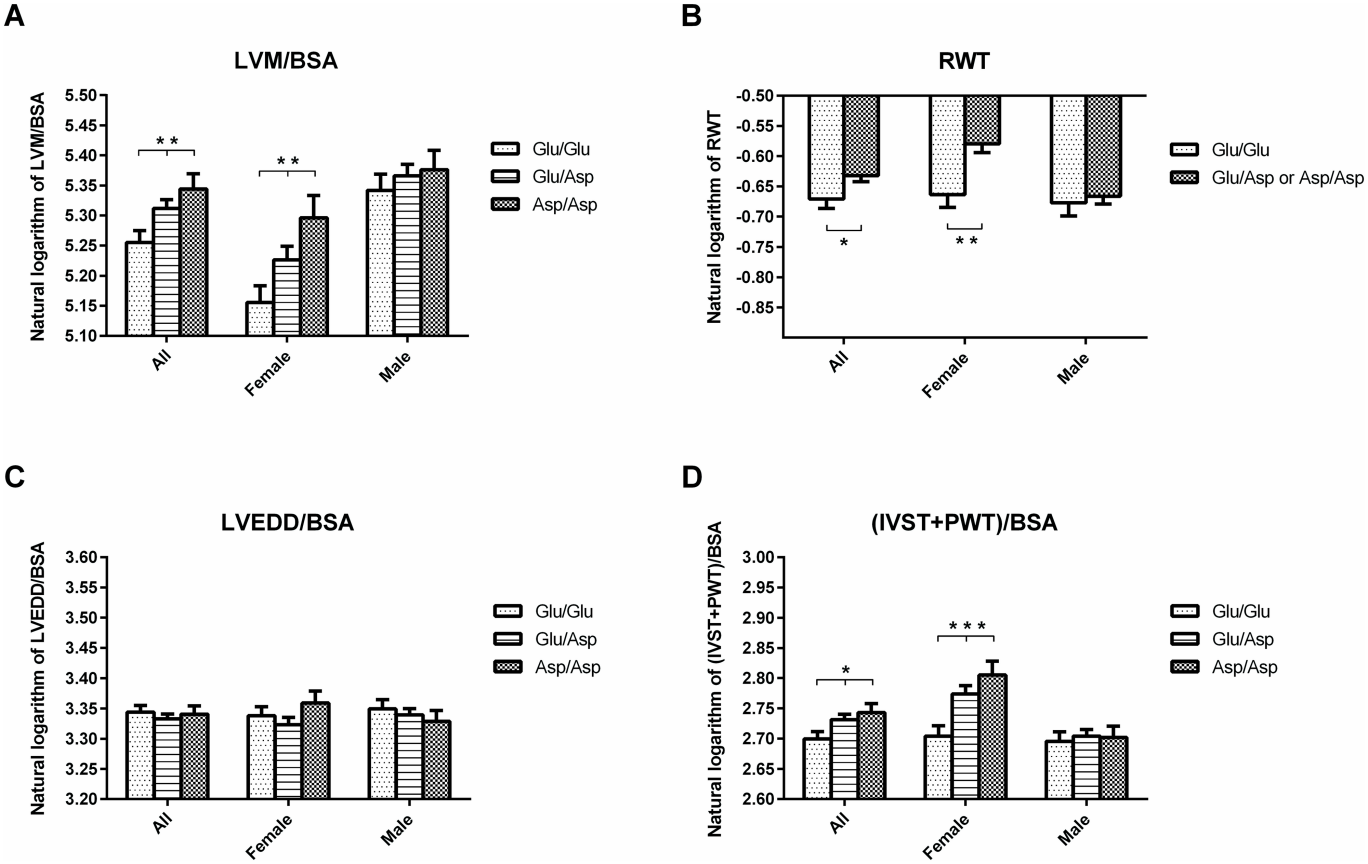
Echocardiographic parameters of left ventricular hypertrophy classified by genotype at rs2296545 and gender. BSA, body surface area; IVST, intraventricular septal thickness in diastole; LVEDD, left ventricular end-diastolic diameter; LVM, left ventricular mass; PWT, posterior wall thickness in diastole; RWT, relative wall thickness; *P < 0.05, **P < 0.005, ***P < 0.0005; crude model; generalized linear models. (A) Association between rs2296545 and natural logarithm of LVM/BSA in the additive genetic model. (B) Association between rs2296545 and natural logarithm of RWT in the dominant genetic model. (C) Natural logarithm of LVEDD/BSA based on rs2296545 in the additive genetic model. (D) Association between rs2296545 and natural logarithm of (IVST+PWT)/BSA in the additive genetic model.

The gender-specific analyses showed a significant association of the Asp37 variant of rs2296545 (Table 2 and Fig 1) with high LVM/BSA in the crude model (mean: 173.3 for Glu/Glu variant, 186.0 for Glu/Asp, and 199.5 for Asp/Asp, p = 0.0021) and a suggestive association in the full model (p = 0.0551) in females. No significant associations were found among the male patients.

### Associations between rs2296545 polymorphism and echocardiographic parameters

The subsequent detailed analysis of the echocardiographic parameters in females (Table 3) revealed a significant association of the Asp37 variant of rs2296545 with high LVM/height (p = 0.0023), LVM/height^2.7^ (p = 0.0005) in the crude model and suggestive association in full model (p = 0.0586, p = 0.0167, respectively) in the additive genetic model. Furthermore the Asp37 variant in the same genetic model was strongly associated (p value range from 3.48 x 10^−05^ to 0.0008) with higher thickness of the cardiac walls regardless of indexation method and the associations remained significant after adjustment for clinical factors in the full model. The association of the Asp37 variant with higher RWT was better described by dominant genetic model (p = 0.0012 and p = 0.0097, for crude and full model respectively).

**Table 3:** Mean values of the echocardiographic parameters based on rs2296545 genotypes in females.

	Crude				Adjusted*			
	Glu/Glu (N = 88)	Glu/Asp (N = 134)	Asp/Asp (N = 48)	P_ADD_	Glu/Glu (N = 87)	Glu/Asp (N = 134)	Asp/Asp (N = 48)	P_ADD_
log LVM/BSA, g/m^2^	5.155 (0.027)	5.226 (0.022)	5.296 (0.037)	0.0021	5.188 (0.025)	5.215 (0.020)	5.272 (0.033)	0.0551
log LVM/height, g/m	5.221 (0.027)	5.292 (0.022)	5.359 (0.037)	0.0023	5.252 (0.025)	5.281 (0.020)	5.334 (0.033)	0.0586
log LVM/height^2.7^, g/m^2.7^	4.411 (0.028)	4.492 (0.023)	4.579 (0.038)	0.0005	4.445 (0.026)	4.481 (0.020)	4.553 (0.034)	0.0167
log LVEDD, mm	3.879 (0.013)	3.859 (0.010)	3.880 (0.017)	0.7889	3.881 (0.012)	3.858 (0.009)	3.881 (0.016)	0.7635
log LVEDD/BSA, mm/m^2^	3.337 (0.014)	3.323 (0.012)	3.358 (0.020)	0.5936	3.341 (0.013)	3.321 (0.010)	3.359 (0.018)	0.6546
log LVEDD/height, mm/m	3.403 (0.013)	3.389 (0.010)	3.421 (0.017)	0.6182	3.406 (0.012)	3.387 (0.009)	3.422 (0.016)	0.6690
log IVST, mm	2.580 (0.016)	2.640 (0.013)	2.664 (0.021)	0.0008	2.599 (0.014)	2.635 (0.011)	2.647 (0.019)	0.0312
log IVST/BSA, mm/m^2^	2.038 (0.018)	2.103 (0.014)	2.143 (0.024)	0.0003	2.059 (0.016)	2.098 (0.013)	2.125 (0.022)	0.0143
log IVST/height, mm/m	2.104 (0.016)	2.170 (0.013)	2.206 (0.022)	0.0001	2.124 (0.014)	2.164 (0.011)	2.187 (0.019)	0.0074
log PWT, mm	2.523 (0.014)	2.590 (0.011)	2.598 (0.020)	0.0007	2.541 (0.013)	2.584 (0.010)	2.581 (0.017)	0.0323
log PWT/BSA, mm/m^2^	1.981 (0.017)	2.053 (0.014)	2.076 (0.024)	0.0005	2.001 (0.016)	2.047 (0.013)	2.059 (0.022)	0.0219
log PWT/height, mm/m	2.046 (0.015)	2.119 (0.012)	2.139 (0.021)	0.0001	2.066 (0.013)	2.114 (0.011)	2.122 (0.018)	0.0079
log (IVST+PWT), mm	3.246 (0.014)	3.310 (0.011)	3.326 (0.019)	0.0003	3.264 (0.012)	3.305 (0.010)	3.309 (0.016)	0.0175
log (IVST+PWT)/BSA, mm/m^2^	2.704 (0.017)	2.773 (0.013)	2.805 (0.023)	0.0002	2.724 (0.015)	2.768 (0.012)	2.787 (0.020)	0.0109
log (IVST+PWT)/height, mm/m	2.770 (0.015)	2.840 (0.012)	2.868 (0.020)	3.48 x 10^−05^	2.789 (0.013)	2.834 (0.010)	2.850 (0.017)	0.0035
	Glu/Glu (N = 88)	Glu/Asp or Asp/Asp (N = 182)		P_DOM_	Glu/Glu (N = 87)	Glu/Asp or Asp/Asp (N = 182)		P_DOM_
log RWT	-0.664 (0.021)	-0.580 (0.015)		0.0012	-0.647 (0.019)	-0.587 (0.013)		0.0097

Data are expressed as the mean and (standard error). N, number of individuals; BSA, body surface area; IVST, intraventricular septal thickness in diastole; LVEDD, left ventricular end-diastolic diameter; LVM, left ventricular mass; PWT, posterior wall thickness in diastole; RWT, relative wall thickness; log, natural logarithm; P_ADD_, p value for additive model; P_DOM_, p value for dominant model;

* adjusted for age, ejection fraction, maximal aortic gradient.

No significant associations were found among the male patients except borderline association of the Asp37 variant with high IVST (p = 0.0499) (S1 Table).

In females, rs2296545 x CAD interaction included in crude and full models was not significant for any of the echocardiographic parameters.

### Homogeneity of the effect of rs2296545 on LVM/BSA and RWT across clinically relevant subgroups

To test for homogeneity of the effect of rs2296545 on LVM/BSA and RWT across clinically relevant subgroups, we divided the cohort into two groups. The first group consisted of patients without significant coronary artery disease or hypertension, with EF > = 50% and transaortic maximum velocity > = 4.0 (symptomatic severe high-gradient AS) which accounted for 40% of patients (n = 243). The remaining patients formed the second group (n = 362) which included the other subgroups: symptomatic severe high-gradient AS with at least one of the following characteristic: reduced EF, hypertension, significant coronary artery disease (n = 244); symptomatic severe low-flow/low-gradient AS with reduced EF (n = 25); symptomatic severe low-gradient AS with normal EF or paradoxical low-flow severe AS (n = 24). Belonging to the first (1) or the second (0) group was used as an interaction term with rs2296545. In females, the tested interaction was not significant for LVM/BSA (p = 0.47 and p = 0.32 for crude and full model, respectively) in the additive genetic model as well as for RWT (p = 0.14 and p = 0.38, for crude and full model respectively) in the dominant genetic model. The above interactions were not significant in males as well. These results allowed us to conclude that the effect of rs2296545 on LVM/BSA or RWT is not different in the two groups.

### Transcription factor binding site analysis

C allele on the reference DNA strand, which corresponds to Asp at amino acid residue 37, increased the predicted DNA binding affinity of the following transcription factors: ATF, CBF2, CREB, GATA-1, Nkx5-1, PEBP, TGA1, TREB-1, but decreased binding probability for CACCC-binding factor, COUP-TF2, GCN4, HNF-4, Nrf-2.

## Discussion

In the present study of 657 patients with severe AS, we found an association of Asp37 variant at amino acid residue 37 of renalase protein with left ventricular hypertrophy in female subjects in an additive genetic model. In another study reported by Farzaneh-Far et al, Asp37 variant was associated with higher cardiac mass in recessive genetic model in patients with stable coronary disease[16]. However, that study included 84.7% male subjects (compared to our population which was 57.7% male, and unfortunately gender-specific data are not shown. Differences in LVH based on rs2296545 genotypes in our population can be detected in the entire group but they are solely caused by female data. Also, our study subjects with significant CAD comprised a substantially smaller group (21.0%), but results in patients with or without CAD are similar. A common factor between the CAD population and AS patients is a hypoxia of ventricular cardiomyocytes. Additionally, pressure overload due to diminished aortic valve area greatly potentiates the effect of concomitant pathological stimuli. This effect is reflected by LVH since mean LVM/BSA in our group doubled the mean value in the CAD population described by Farzaneh-Far et al. As described later, one can hypothesize that Asp37 variant effect is pronounced under hypoxia and pressure overload condition when glycolysis is increased and renalase becomes more important to preserve primary metabolism. Thus, 2 Asp37 variants were needed to produce an effect on LVM/BSA in the CAD population, but in AS the effect was evident with each dose of Asp37 variant. These 2 studies included different populations yet an association between Asp37 variant and left ventricular hypertrophy was confirmed, suggesting a potential causal relationship.

In women Asp37 variant was strongly associated with thickness of the posterior wall and interventricular septum as well as LVM, regardless of indexation method. Women heterozygous or homozygous for Asp37 had also higher RWT. In males, only a weak association of IVST with renalase Asp37 variant was observed. In previous studies we also reported sex-related differences in the association between common genetic polymorphism of the chymase CMA1 gene[17] and ACE gene[28] with left ventricular mass.

There are significant sex differences in the response to pressure overload. Females develop more concentric hypertrophy with greater RWT, IVST/BSA, PWT/BSA and a better systolic function and higher transvalvular gradients than males as we previously described using a large number of patients with AS[17]. Observations have shown that after valve replacement, LVH reversed more frequently and faster in women than in men[29].

Hypertrophic response to pressure overload initially comprises an increase in cardiomyocyte size and amount of contractile proteins without cellular proliferation. Hypertrophy later becomes maladaptive with an increased formation of fibrotic tissue, resulting in dilatation and impaired function of the left ventricle[29, 30]. Differences in the hypertrophic response between the sexes may be due to the different molecular mechanism involved. Regitz-Zagrosek et al.[31] performed genome-wide expression profiling of myocardial samples from subjects undergoing aortic valve replacement. Transcriptome analysis revealed that fibrosis-related genes were upregulated in overloaded male ventricles, while extracellular matrix-related and inflammatory genes were downregulated in female samples. These gene expression patterns were associated with the tissue content of collagen and inflammatory cells detected with immunostaining. It is plausible that sex-related response to pressure overload may be modulated by ischemia.

Renalase circulates in peripheral blood and is strongly expressed in the heart and kidney. There are no data on sex-related differences in these organs and circulating levels of renalase. Until now, renalase was considered a kidney-derived enzyme degrading catecholamines. In experimental models lower expression of the renalase gene was associated with higher blood pressure and administration of recombinant protein had a hypotensive effect in Dahl salt-sensitive rats[7]. Nephrectomy induces a fall in plasma levels of renalase. After 5/6 nephrectomy male rats develop higher blood pressure and left ventricular hypertrophy with increased content of collagen. These changes were significantly ameliorated by administration of renalase [32]. In humans, renalase blood levels correlate with glomerular filtration rate and after bilateral nephrectomy patients have no renalase expression and no detectable plasma level and activity[6]. Many studies reported a correlation between plasma renalase level and systolic blood pressure, age and presence of heart failure (reviewed in[33]). Based on the postulated mechanism, renalase was considered an enzyme which protects tissues in the state of adrenergic activation and excess of catecholamines.

Catecholamines promote hypertrophy of cardiac myocytes and hyperplasia of cardiac fibroblasts and they can arrive to the heart from the periphery or can be synthesized locally[34]. Both local release of norepinephrine from cardiac sympathetic nerves and epinephrine from adrenal glands could be involved in the left ventricle remodeling in AS. Chronic pressure overload induces adrenal hypertrophy and increases epinephrine synthesis[35]. Elevated cardiac epinephrine content and plasma norepinephrine concentrations were also described in rats after transverse aortic constriction[36].

In our study Glu37Asp polymorphism was strongly associated with the thickness of the left ventricle walls but not with its internal dimension. Therefore adrenergic stimulation of cardiomyocytes seems more important for this pattern of LVH than catecholamine-related activation of cardiac fibroblasts, resulting not only in fibrosis but also in collagen degradation in mice[37].

Recent findings question the role of renalase as a catecholamine degrading enzyme. Studies by Moran et al suggest that the metabolic function of renalase is to oxidize isomeric NAD(P)H molecules 2- and 6-dihydroNAD (which are potent inhibitors of primary metabolism dehydrogenases) to β-NAD(P)+ and thereby eliminating potential injury to intracellular respiratory activity[9, 38].

Among the three dehydrogenases tested, both 2- and 6-dihydroNAD had high affinity for lactate dehydrogenase (LDH) but no significant affinity for lipoamide dehydrogenase. 6-dihydroNAD had exceedingly high affinity for malate dehydrogenase (MDH) but 2-dihydroNAD had only a modest inhibitory effect for this enzyme[9]. MDH is an important enzyme for mitochondrial oxidative metabolism. LDH converts pyruvate to lactate by utilizing the NADH generated from glycolysis. This is an efficient manner to oxidize cytosolic NADH to NAD+, which allows for an increase in the glycolytic rate as the regeneration of NAD+ is a rate-limiting step for glycolysis[39]. It is a widely held belief that hypertrophied and failing hearts demonstrate increased glycolysis[40], and lactate dehydrogenase activity has been shown to increase in experimental models of cardiac hypertrophy[41, 42]. Therefore the primary metabolism appears to be a switch from mitochondrial oxidative metabolism to an increase in glucose uptake and glycolysis[43]. Interestingly, a gender-related difference in glycolysis was found in hypertrophied rat hearts subjected to ischemia[44]. We can hypothesize that lower renalase availability could cause an increase in 2- and 6-dihydroNAD and subsequently greater inhibition of LDH which will lead to decreased concentration of NAD+ and increased NADH. The data from ischemia–reperfusion injury in vivo model where the myocardial levels of NAD+ and ATP decreased when renalase was knocked down by siRNA[45] seem to support the above thesis. Agonist-induced cardiac hypertrophy is associated with loss of intracellular levels of NAD but not with exercise induced physiologic hypertrophy. Exogenous addition of NAD was capable of maintaining intracellular levels of NAD and blocking the agonist-induced cardiac hypertrophic response in vitro as well as in vivo. NAD treatment mediated through activation of SIRT3 blocked the activation of pro-hypertrophic Akt1 signaling and augmented the activity of anti-hypertrophic LKB1-AMPK signaling in the heart, preventing subsequent induction of mTOR-mediated protein synthesis[46].

The plasma membrane calcium/calmodulin dependent ATPase (PMCA4b)is an extracellular receptor of renalase. The action mediated through this receptor promotes cell survival and defense against cisplatin toxicity through phosphorylation of p38 (MAPK) in human kidney embryonic cell line (HK-2). This was established using PMCA4b-targeting siRNAs and Caloxin1b, a peptide inhibitor of PMCA4b[10]. PMCA4b gene expression in the renalase knockout mouse was 11.4 fold lower than in wild type (WT) control; corresponding PMCA4b protein expression in the renalase knockout was 63.5±7.5% lower than in WT[10]. It was found that PMCA4b affects pressure-overload hypertrophy in a cell specific manner[11, 47].

Interestingly, PMCA4b is involved in the regulation of catecholamine secretion by the rat adrenal medulla cells[48] and is itself regulated by 17β-estradiol[49].

It was initially suggested that a conserved amino acid change at residue 37 (glutamic to aspartic acid) is located within the flavin-adenine dinucleotide-binding domain and Asp37 variant is associated with a 24-fold decrease in affinity for NADH, a 2.3-fold reduction in maximal renalase enzymatic activity and a lower catecholamine degradation rate[16]. Recently, direct catecholamine catabolism by renalase[38] and the functionality of the FAD domain[8] were questioned. Moreover the 20 amino acid renalase peptide (RP-220, aa 220–239), which is conserved in all known isoforms but is devoid of any detectable oxidase activity and lacks the FAD domain, was as effective as intact renalase protein at protecting HK-2 cells and WT mice against toxic and ischemic injury[50] and acts through PMCA4b[10]. The question then arises as to the functional meaning of the Glu37Asp polymorphism for the RNLS gene itself and for the pathophysiology of pressure overload hypertrophy. Binding of transcription factors to regulatory DNA regions in place of canonical nucleosomes triggers chromatin remodeling and results in nuclease hypersensitivity[51]. Interestingly, according to data available from ENCODE consortium, rs2296545 polymorphism is located in the DNase I–hypersensitive site[19], which is important for gene expression regulation.

Approximately 15% of human codons are dual-use codons (“duons”) that simultaneously specify both amino acids and transcription factor recognition sites[52]. Single-nucleotide variants within duons can cause not only amino acid change but can also directly alter transcription factor binding[19]. The computational prediction revealed that rs2296545 affects few transcription factor binding sites including CREB[53] and ATF[54]–known factors involved in pathological hypertrophic response of the myocardium–and could influence RNLS gene expression in this mechanism.

In analysis of over 114 cell and tissue types and states sampled from 166 individuals, rs2296545 did not directly influence the chromatin architecture of individual regulatory regions in an allele-specific fashion[19], but only healthy fetal and adult heart tissues were represented among the analyzed samples. In AS, left ventricular tissue is subjected to pressure overload and hypoxia which induce a specific set of transcription factors. Some of those factors in which transcription factor binding sites are affected by rs2296545 may be absent in normal cardiac tissue. It is postulated that each cell/tissue contains a specific set of gene regulators[51] out of the known 1400 human transcription factors[55] and this could be especially true for pathological tissue.

There are some limitations in our study which should be taken into consideration. First, we did not investigate the functional consequences of the polymorphism in individual patients at the left ventricular tissue level, such as RNA and protein concentration. Functional studies using animal models of pressure overload hypertrophy are required to elucidate the molecular mechanism through which rs2296545 affects the RNLS promoter activity in cardiac cells subjected to pressure overload, cardiac catecholamine concentration, cardiac primary metabolism, and finally cardiac hypertrophy in gender-related manner. Second, the present study consisted solely of Caucasians and the results may not apply to other populations. Third, the AS patient population is heterogeneous[56] but the effect of rs2296545 on LVM/BSA or RWT in our study was not different in the group consisting of patients with symptomatic severe high-gradient AS without significant coronary artery disease or hypertension, compared to the group formed by the remaining patients. Fourth, due to the extreme hypertrophy present in some of our patients, one could question its etiology differences, such as hypertrophic cardiomyopathy (HCM). We did not perform exome sequencing in patients with extreme values of LVM/BSA to rule out HCM, but with the prevalence of 1/200 to 1/500 in the general population, HCM would have only a small effect on our results. Moreover all patients with the LVM/BSA above 90 quantile (LVM/BSA> 282.2 g/m2) reduced LVM after surgery, which makes diagnosis of HCM very unlikely in these patients.

In conclusion, we have found the association of the renalase Glu37Asp polymorphism with left ventricle hypertrophy in large group of females with AS. Our data suggest that renalase plays a role in the hypertrophic response to pressure overload, however, the exact mechanism requires further investigation.

## Supporting information

S1 Table Mean values of the echocardiographic parameters based on rs2296545 genotypes in males. (PDF)

S2 Table Raw data. (XLSX)
