# Supplementary material for: Functional polymorphism of the renalase gene is associated with cardiac hypertrophy in female patients with aortic stenosis
Source: PLoS One. 2017 Oct 24;12(10):e0186729. doi: 10.1371/journal.pone.0186729 (PMC5655536; doi:10.1371/journal.pone.0186729)
Supplement: S1 Table — (PDF) [file pone.0186729.s001.pdf]

S1 Table. Mean values of the echocardiographic parameters based on rs2296545 genotypes in males.

	Crude				Adjusted*			
	Glu/Glu (N=101)	Glu/Asp (N=210)	Asp/Asp (N=72)	P_{ADD}	Glu/Glu (N=100)	Glu/Asp (N=206)	Asp/Asp (N=72)	P_{ADD}
log LVM/BSA, g/m^2	5.341 (0.027)	5.366 (0.018)	5.376 (0.032)	0.3887	5.344 (0.025)	5.370 (0.017)	5.376 (0.029)	0.3905
log LVM/height, g/m	5.425 (0.026)	5.463 (0.018)	5.485 (0.031)	0.1346	5.427 (0.025)	5.468 (0.017)	5.485 (0.029)	0.1197
log LVM/$height^{2.7}$, g/$m^{2.7}$	4.506 (0.027)	4.540 (0.019)	4.557 (0.032)	0.2109	4.507 (0.026)	4.545 (0.018)	4.558 (0.030)	0.1879
log LVEDD, mm	3.973 (0.013)	3.978 (0.009)	3.983 (0.016)	0.6549	3.970 (0.011)	3.978 (0.008)	3.997 (0.013)	0.1460
log LVEDD/BSA, mm/m^2	3.349 (0.015)	3.339 (0.010)	3.328 (0.018)	0.3801	3.346 (0.013)	3.337 (0.009)	3.342 (0.015)	0.8065
log LVEDD/height, mm/m	3.433 (0.013)	3.436 (0.009)	3.437 (0.016)	0.8234	3.429 (0.011)	3.435 (0.007)	3.451 (0.013)	0.2154
log IVST, mm	2.647 (0.016)	2.679 (0.011)	2.695 (0.019)	0.0499	2.654 (0.014)	2.683 (0.010)	2.677 (0.017)	0.2413
log IVST/BSA, mm/m^2	2.022 (0.017)	2.039 (0.012)	2.040 (0.020)	0.4857	2.030 (0.016)	2.043 (0.011)	2.022 (0.019)	0.8527
log IVST/height, mm/m	2.106 (0.016)	2.136 (0.011)	2.149 (0.019)	0.0891	2.113 (0.015)	2.141 (0.010)	2.131 (0.018)	0.3522
log PWT, mm	2.603 (0.013)	2.618 (0.009)	2.628 (0.016)	0.2428	2.607 (0.012)	2.622 (0.008)	2.613 (0.014)	0.6953
log PWT/BSA, mm/m^2	1.978 (0.015)	1.978 (0.010)	1.973 (0.018)	0.8299	1.984 (0.014)	1.982 (0.009)	1.959 (0.016)	0.2874
log PWT/height, mm/m	2.062 (0.014)	2.075 (0.010)	2.082 (0.017)	0.3597	2.067 (0.013)	2.079 (0.009)	2.068 (0.015)	0.8672
log (IVST+PWT), mm	3.320 (0.014)	3.344 (0.009)	3.356 (0.016)	0.0840	3.326 (0.012)	3.348 (0.008)	3.340 (0.014)	0.3681
log (IVST+PWT)/BSA, mm/m^2	2.695 (0.015)	2.704 (0.010)	2.701 (0.018)	0.7579	2.702 (0.014)	2.708 (0.009)	2.686 (0.016)	0.5335
log (IVST+PWT)/height, mm/m	2.779 (0.014)	2.801 (0.010)	2.811 (0.017)	0.1485	2.785 (0.013)	2.805 (0.009)	2.795 (0.015)	0.5222

	Glu/Glu (N=101)	Glu/Asp or Asp/Asp (N=282)	P_{DOM}	Glu/Glu (N=100)	Glu/Asp or Asp/Asp (N=278)	P_{DOM}
log RWT	-0.677 (0.022)	-0.666 (0.013)	0.6588	-0.669 (0.018)	-0.670 (0.011)	0.9688

Data are expressed as the mean and (standard error). N, number of individuals; BSA, body surface area; IVST, intraventricular septal thickness in diastole; LVEDD, left ventricular end-diastolic diameter; LVM, left ventricular mass; PWT, posterior wall thickness in diastole; RWT, relative wall thickness; log, natural logarithm; P_{ADD}, p value for additive model; P_{DOM}, p value for dominant model; * adjusted for age, ejection fraction, maximal aortic gradient,
